# Supplementary material for: Change in the Electronic Structure of the Cobalt(II) Ion in a One-Dimensional Polymer with Flexible Linkers Induced by a Structural Phase Transition
Source: Int J Mol Sci. 2022 Dec 22;24(1):215. doi: 10.3390/ijms24010215 (PMC9820815; doi:10.3390/ijms24010215)
Supplement: Supplementary file 1 [file ijms-24-00215-s001.zip › ijms-2104629-supplementary.pdf]

Electronic supplementary materials for manuscript

Change in the Electronic Structure of the Cobalt(II) Ion in a One-Dimensional Polymer with Flexible Linkers Induced by a Structural Phase Transition

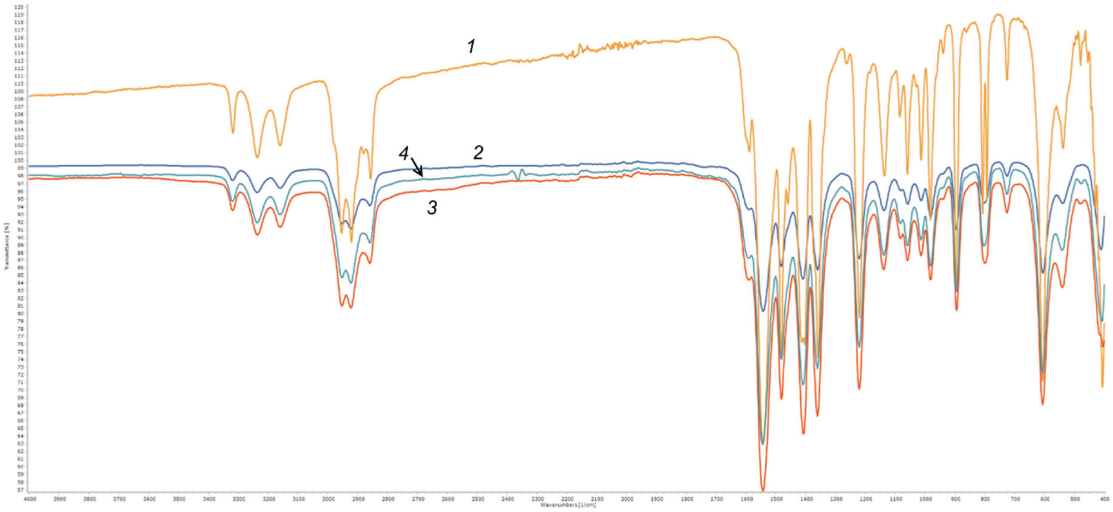

Figure S1. IR spectra for **1** (*1* – initial sample as synthesized, *2* – sample after one-month exposure on air at RT, *3* – sample after 21 h in liquid nitrogen bath, *4* - sample after 5 h in ice bath at 173-183 K).

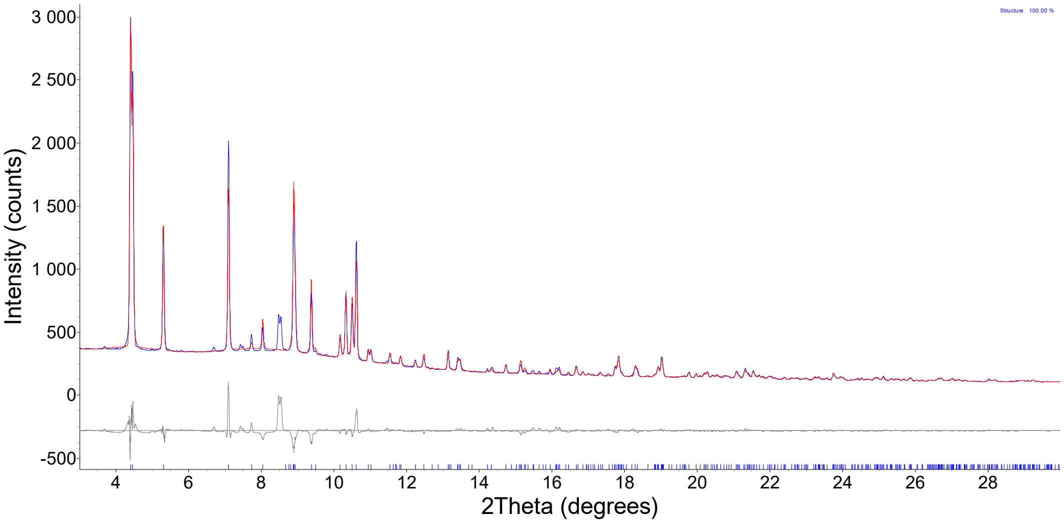

Figure S2. Description of the diffractogram by the Rietveld method using the structure β-**1** (red curve - calculated, blue curve - experiment, gray curve - difference line), R_{wp} = 5.9% (λ = 1.54 Å, at room temperature).

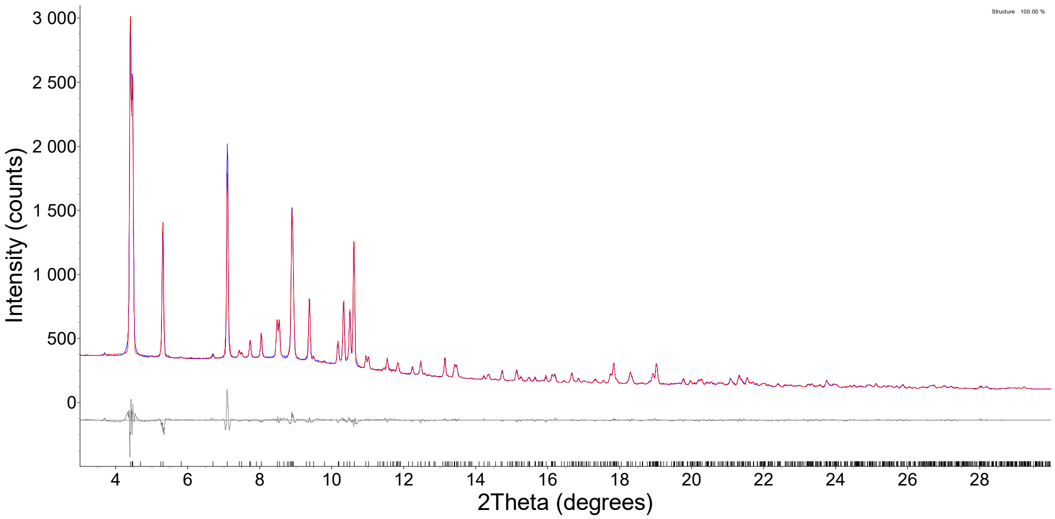

Figure S3. Description of the diffractogram by the Rietveld method using the structure α-**1**, R_{wp} = 2.7% (λ = 1.54 Å, at room temperature).

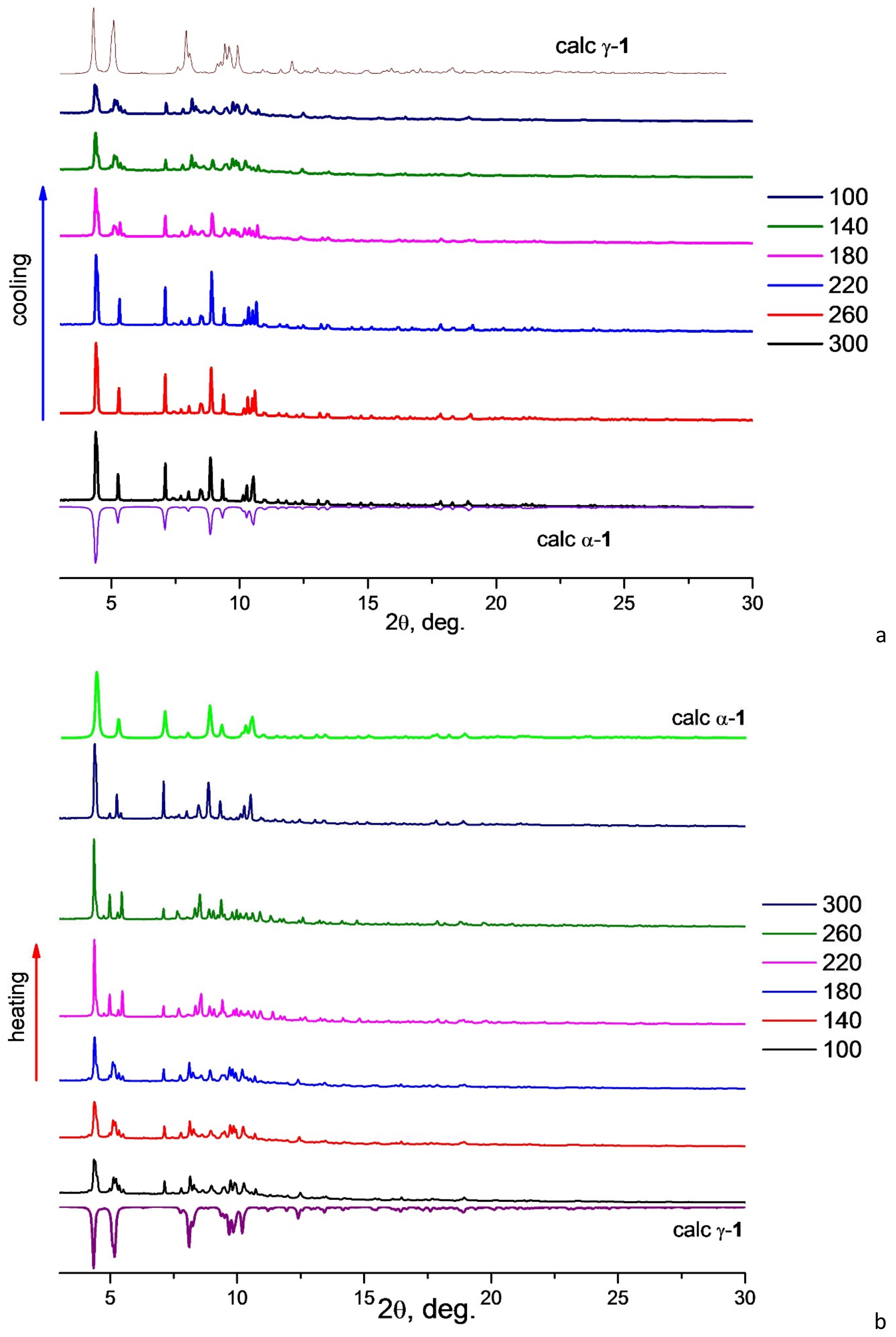

Figure S4. Variable-temperature PXRD measurements of **1** from 300 to 100 K (a) and from 100 to 300 K (b) (λ = 0.74 Å).

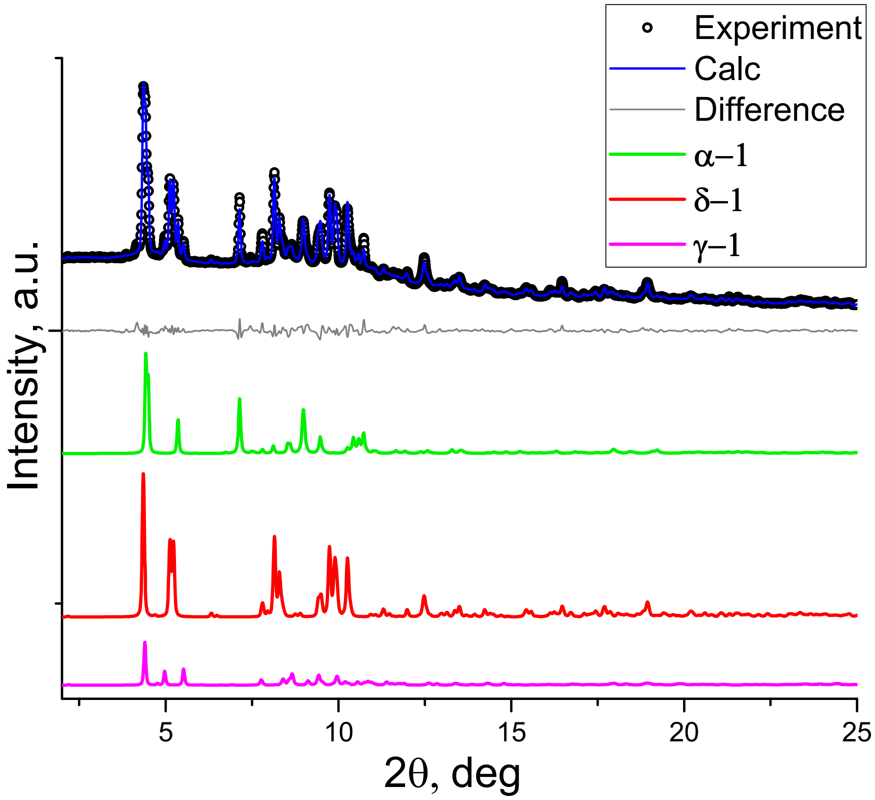

Figure S5. Description of the diffractogram of **1** obtained at 100 K (λ = 0.74 Å).

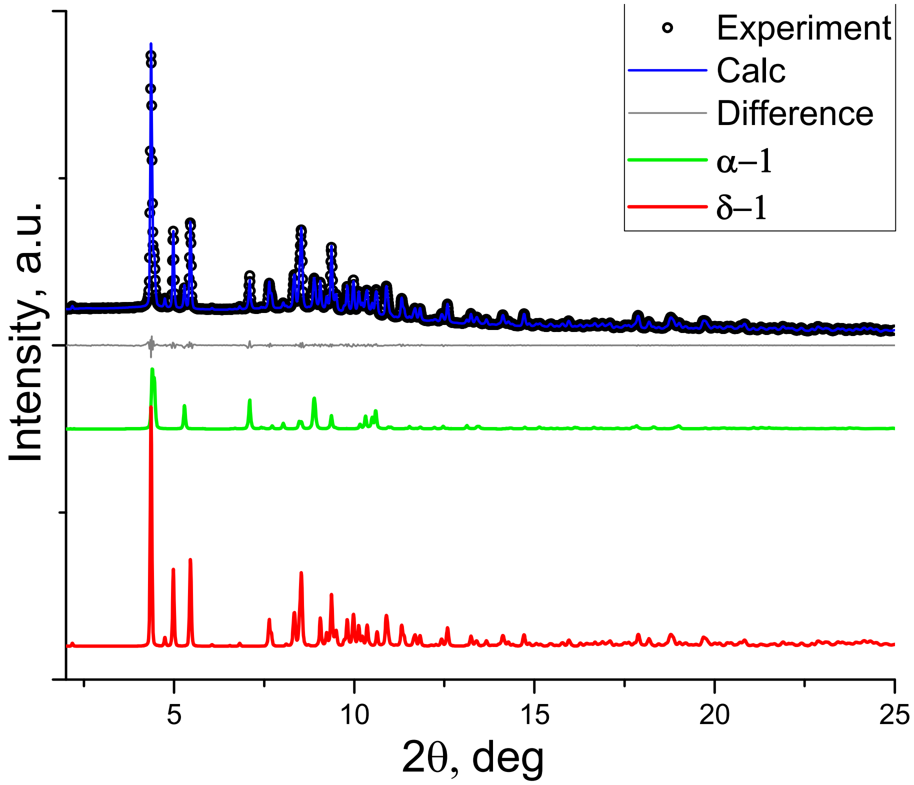

Figure S6. Description of the diffractogram of **1** obtained at 260 K (heating) (λ = 0.74 Å).

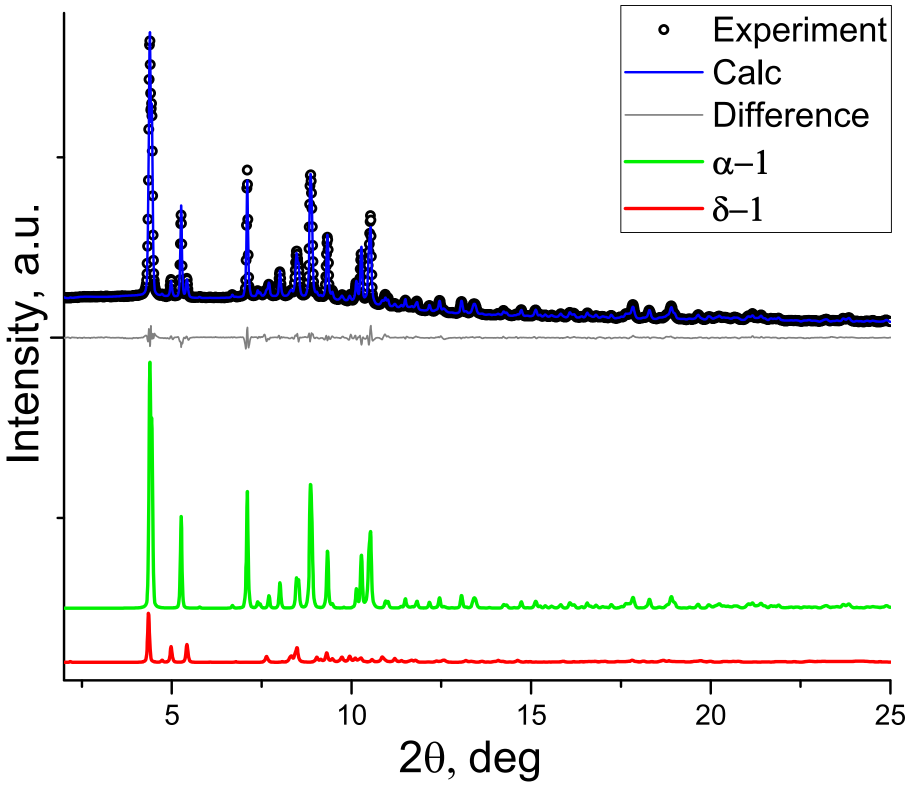

Figure S7. Description of the diffractogram of **1** obtained at 300 K (final) (λ = 0.74 Å).

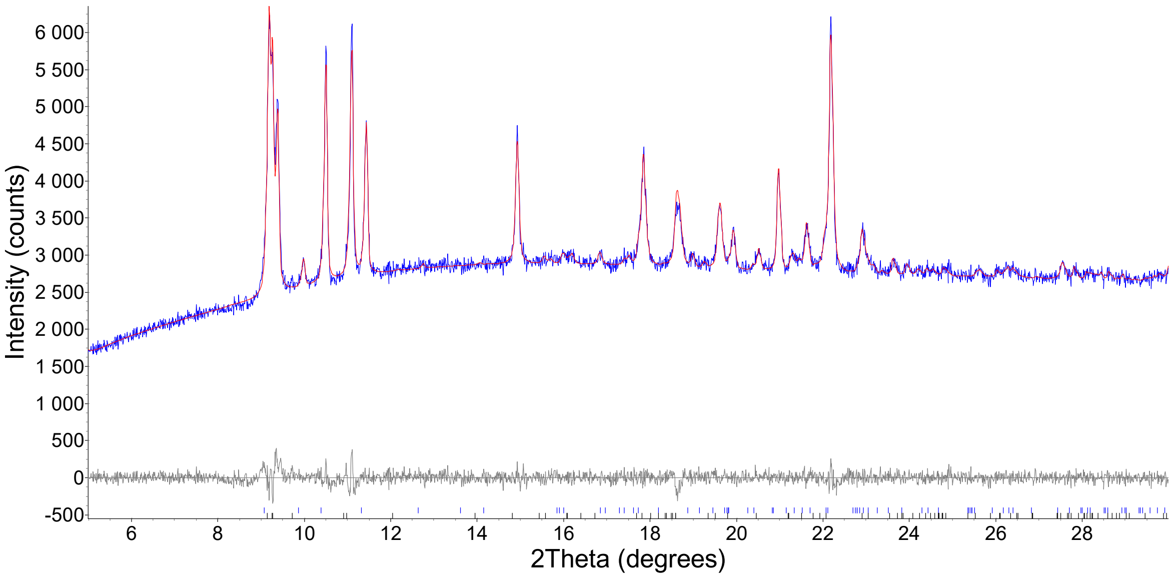

Figure S8. X-ray diffraction patterns of sample **1** (λ = 1.54 Å, at room temperature) held at 170-180 K (exposure 5 hours). The blue vertical ticks show the position of the peaks of the new low-temperature phase δ-**1**, black ticks indicated positions of α-**1**.

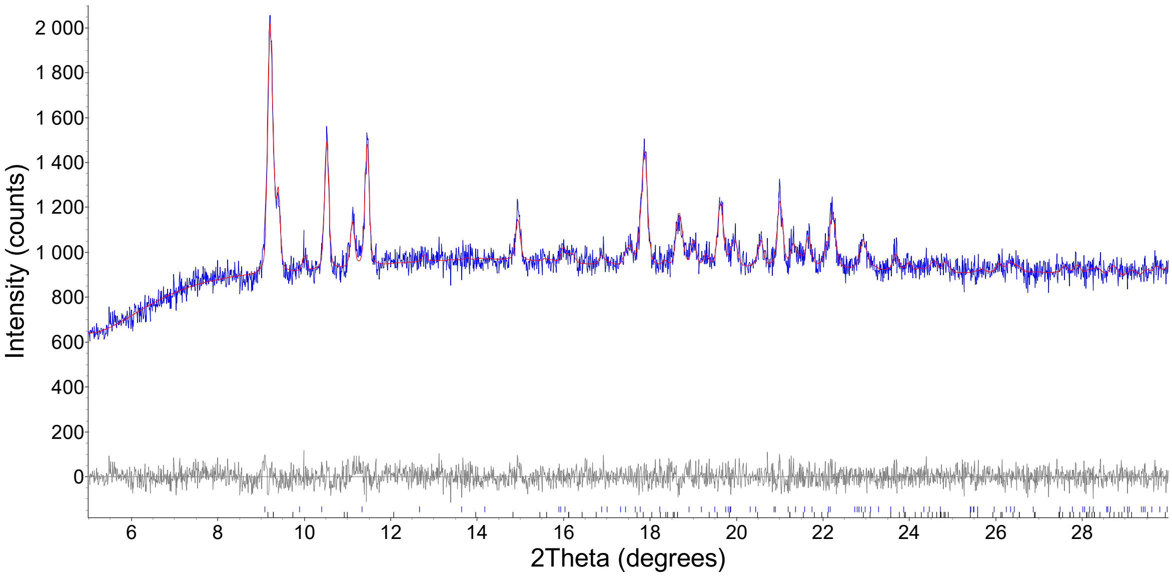

Figure S9. X-ray diffraction patterns of sample **1** (λ = 1.54 Å, at room temperature) held at 77 K (exposure 5 hours). The blue vertical ticks show the position of the peaks of the new low-temperature phase δ-**1**, black ticks indicated positions of α-**1**.

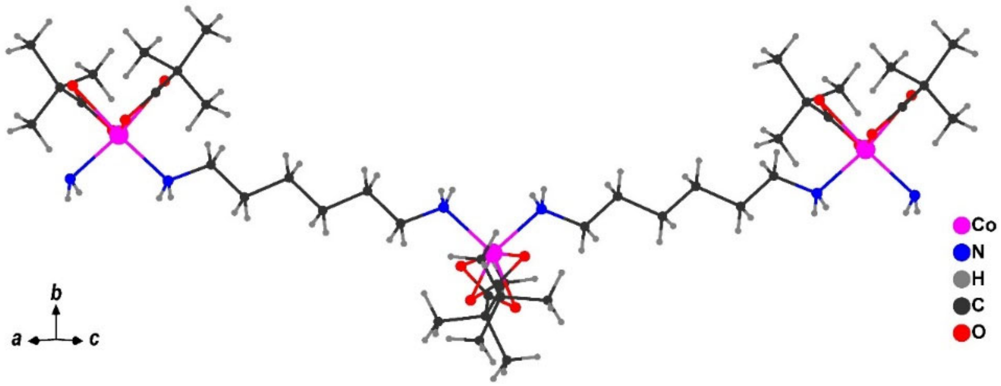

Figure S10. The structure of polymeric chain in β-**1**.

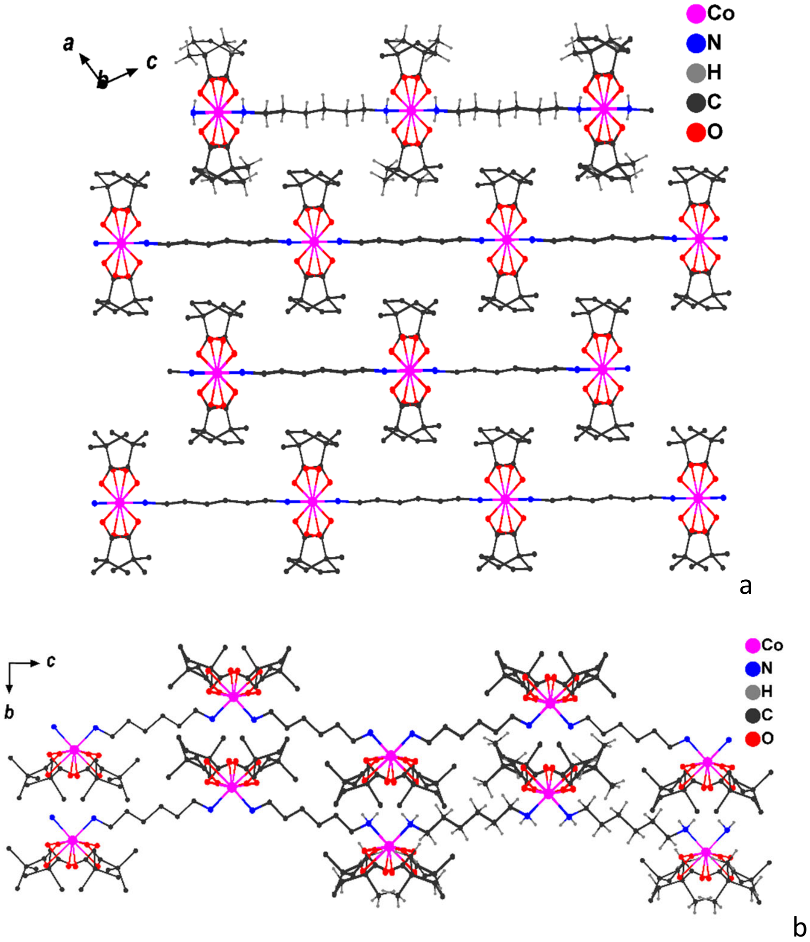

Figure S11. The fragment of packing for β-**1**.

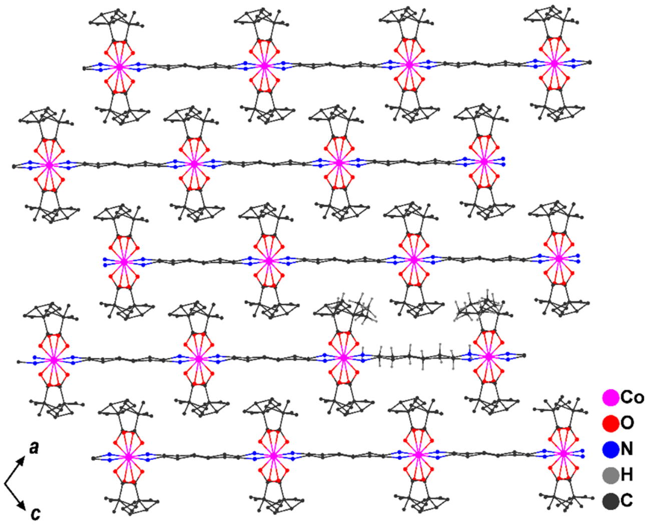

Figure S12. The fragment of packing for γ-**1**.

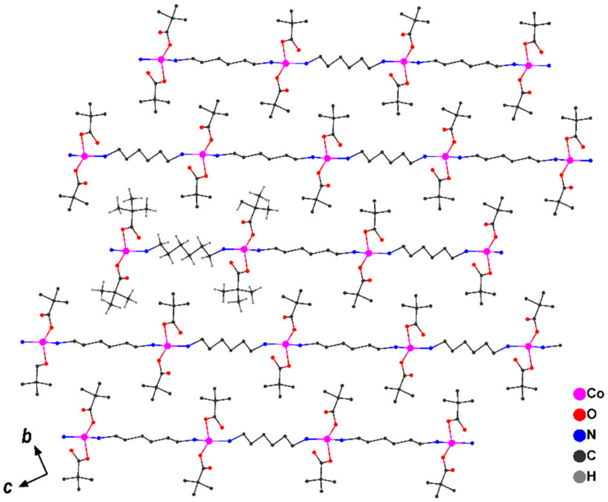

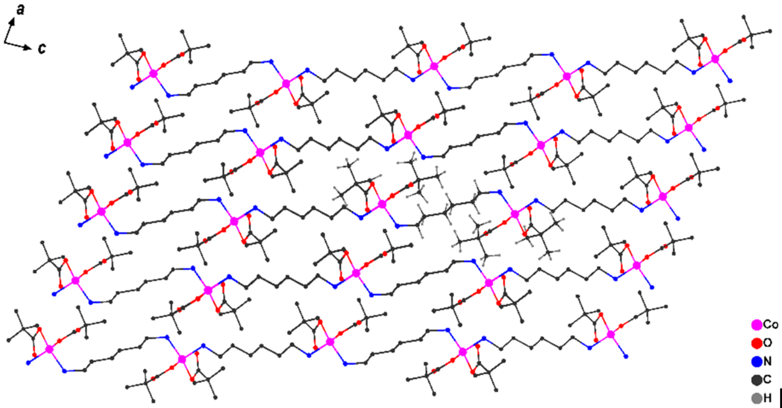

Figure S13. The fragment of packing for γ-**1**.

Table S1. Continuous Shape Measures (CShM) values for the potential coordination polyhedron of Co in β-**1**, β-**1** и γ-**1**.

Structure / Polyhedron / Geometry	α-**1**[*]	β-**1**	γ-**1**
CoN_2O_4			
D_{6h}, Hexagon	28.283, 28.134	30.634	-
C_{5v}, Pentagonal pyramid	19.495, 20.059,	18.477	-
O_h, Octahedron	5.909, 5.609	7.095	-
D_{3h}, Trigonal prism	11.752, 12.683	11.888	-
C_{5v}, Johnson pentagonal pyramid	23.485, 24.057	22.443	-
CoN_2O_3			
D_{5h}, Pentagon	-	-	29.725
C_{4v}, Vacant octahedron	-	-	5.041
D_{3h}, Trigonal bipyramid	-	-	4.050
C_{4v}, Spherical square pyramid	-	-	4.783
D_{3h}, Johnson trigonal bipyramid	-	-	5.018
CoN_2O_2			
D_{4h}, Square	-	-	31.745
T_d, Tetrahedron	-	-	1.426
C_{2v}, Seesaw	-	-	5.357
C_{3v}, Vacant trigonal bipyramid	-	-	2.160

[*] For two independent cobalt atoms.

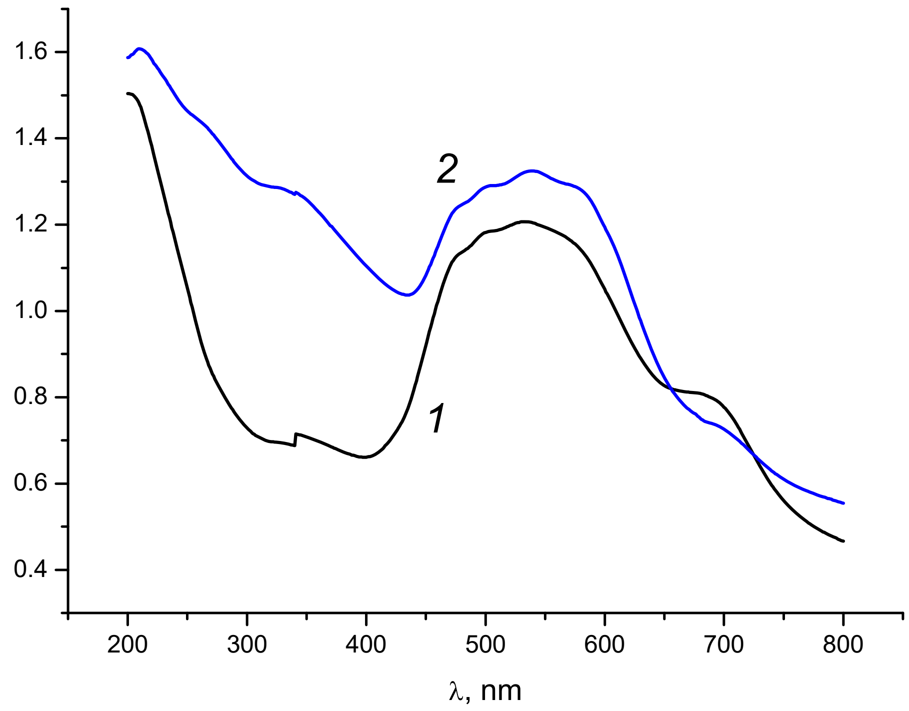

Figure S14. Diffuse reflectance spectra of **1** as synthesized (*1*) and after 5 h in liquid nitrogen bath (*2*).

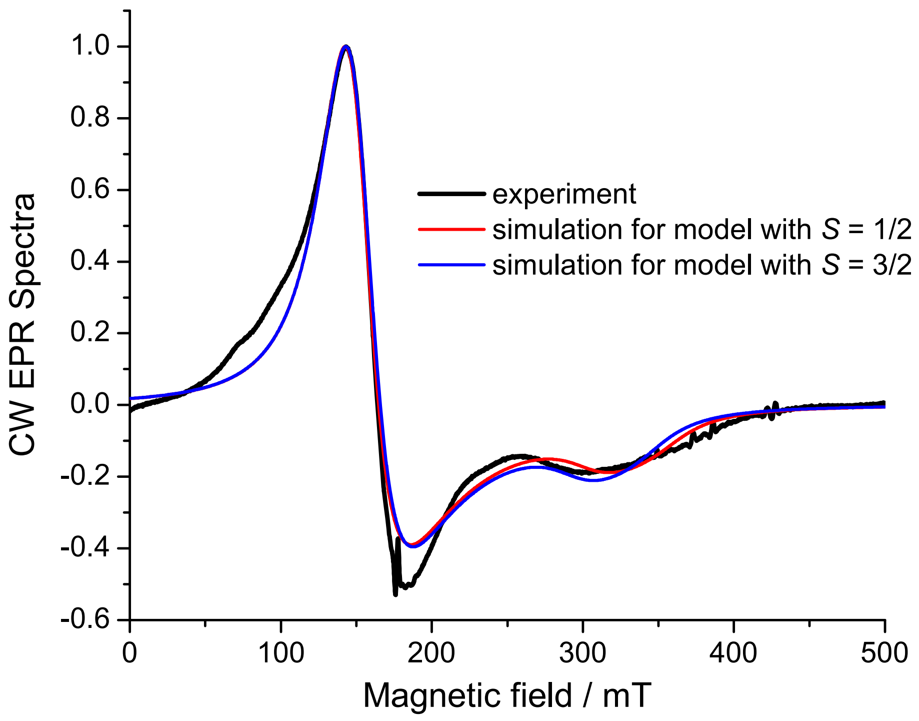

Figure S15. X-band EPR spectrum for **1** (polycrystalline sample, 10 K) and simulation results (see text).

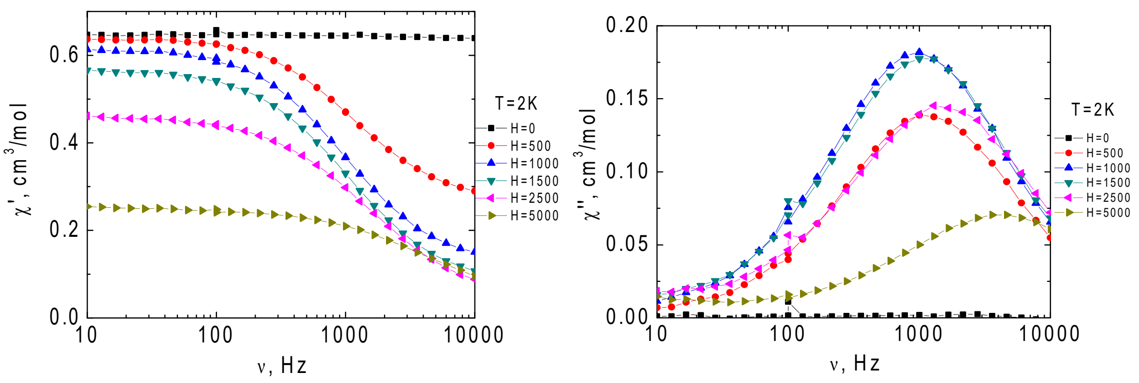

Figure S16. Frequency dependences of the real (χ', *left*) and imaginary (χ'', *right*) parts of the dynamic magnetic susceptibility at different applied magnetic fields at 2 K for **1**.
